# Correlation of TcII discrete typing units with severe chronic Chagas cardiomyopathy in patients from various Brazilian geographic regions

**DOI:** 10.1371/journal.pntd.0010713

**Published:** 2022-12-12

**Authors:** Maykon Tavares de Oliveira, Carlos Alessandro Fuzo, Maria Cláudia da Silva, Eduardo Antônio Donadi, João Santana da Silva, Henrique Turin Moreira, André Schmidt, José Antônio Marin-Neto

**Affiliations:** 1 Department of Internal Medicine, Cardiology Division, Ribeirão Preto Medical School, University of São Paulo, Ribeirão Preto, São Paulo, Brazil; 2 Department of Clinical Analyses, Toxicology and Food Sciences, School of Pharmaceutical Sciences of Ribeirão Preto, University of São Paulo, Ribeirão Preto, São Paulo, Brazil; 3 Fiocruz-Bi-Institutional Translational Medicine Plataform, Ribeirão Preto, São Paulo, Brazil; 4 Department of Internal Medicine, Division of Clinical Immunology, Ribeirão Preto Medical School, University of São Paulo, Ribeirão Preto, São Paulo, Brazil; University of Texas at El Paso, UNITED STATES

## Abstract

**Background:**

Chagas disease (ChD) is caused by *Trypanosoma cruzi*. The genetic structure of the species is divided into seven distinct genetic groups, TcI to TcVI, and Tcbat, which have shown differences in terms of geographic distribution, biological properties, and susceptibility to drugs. However, the association between genetic variability and clinical forms of ChD has not yet been fully elucidated. The predominance of TcII and TcVI discrete typing units (DTUs) (genetic groups) is known to occur in several Brazilian regions and is associated with both the domestic and the wild cycles of ChD. Thus, this study aimed to verify the genotypes of the parasites present in 330 patients with chronic Chagas cardiomyopathy (CCC) from different Brazilian states attended at the Clinical Hospital of the Ribeirão Preto Medical School and to assess the existence of a correlation between the clinical forms with the main cardiovascular risk factors and the genetics of the parasite.

**Methodology Principal findings:**

All patients with CCC were clinically evaluated through anamnesis, physical examination, biochemical tests, 12-lead electrocardiogram, echocardiogram and chest X-ray. Peripheral blood (5 mL) was collected in guanidine/ethylenediaminetetraacetic acid from each patient for DNA extraction and real-time polymerase chain reaction (PCR) for Chagas disease and genotyping of the parasite in the 7 DTUs. Parasite genotyping was performed using conventional multilocus PCR. Samples of only 175 patients were positive after amplification of the specific genes contained in the *T*. *cruzi* genotyping criteria. TcII (64/175), TcVI (9/175), and TcI (3/175) DTUs were predominant, followed by TcII/TcV/TcVI (74/175), and TcII/TcVI (23/175). The TcIII and TcIV DTU´s was detected in only one sample of CCC patients.

**Conclusions/Significance:**

Our data corroborate previous findings, indicating the predominance of the TcII genotype in patients with CCC of Brazilian origin. Moreover, this study pioneered disclosing a direct correlation between the TcII DTU and severe CCC.

## Author summary

*Trypanosoma cruzi* is divided into seven distinct genetic groups (TcI-TcVI) and TcBat. They may be related to several biological parameters, however, the correlation with the clinical forms is not well established. Thus, the work has the important function of identifying the genetic variability of *T*. *cruzi* circulating in different regions of Brazil, and trying to correlate the genetics of the parasite with the clinical manifestations presented by the patients. In this work, we showed differences in the prevalence between infectious DTU’s (genetic groups), and we were able to identify and describe the correlation between TcII DTU and severe chronic Chagas cardiomyopathy (LVEF < 40%). The information generated in this study should impact the planning of more effective public health interventions to improve the health of chagasic patients, control vertical transmission and the treatment of CD in endemic countries.

## Introduction

Chagas disease (ChD) is caused by the hemoflagellate protozoan *Trypanosoma cruzi* [1], which is genetically diverse and has been subdivided into seven genetic lineages or discrete typing units (DTUs), TcI to TcVI, and TcBat [2,3].

According to the World Health Organization, 6–7 million people are chronically infected with *T*. *cruzi* worldwide, and more than 90 million individuals are at risk of infection. It is still one of the infectious and parasitic diseases with the greatest social and economic impact on the American continent because of its high transmission rates, mainly in the Andean countries [4]. Also, a large contingent of infected individuals is widespread in most Latin American countries where the disease is not properly controlled [5,6].

The most frequent and severe clinical manifestations are attributed to heart disease, which includes heart failure, cardiac arrhythmias, thromboembolism, and sudden death. The disease may also cause digestive manifestations that occur in isolation or in association with cardiac manifestations [7–9]. Hypertrophy and dilation of cardiac chambers may occur in advanced cases of Chagas cardiomyopathy and early in the natural history of the disease, regional wall motion abnormalities, including the typical apical aneurysm, usually involving both ventricles are seen [9].

For all characteristic clinical forms of CCC it is now believed that tissue parasitic persistence and the adverse host immune response, associated to main biological parameters of the strains, and host genetics, are the most important factors involved in the pathogenesis of ChD [10].

Knowing that the genetics of the parasite can drastically influence the pathogenesis of the disease, this study aimed to evaluate and correlate the genotype of the infectious agent with the severity of the disease in a sample of patients with CCC from different regions of Brazil.

## Materials and methods

### Ethics statement

This study was approved by the Human Research Ethics Committee of the Clinical Hospital, Ribeirão Preto Medical School, University of São Paulo (FMRP/USP–CAAE:09948419.3.0000.5440). Written informed consent was obtained from all patients.

### Patients

A total of 330 patients managed at the Chagas Disease Outpatient Clinic, Division of Cardiology, Ribeirão Preto Medical School, University of São Paulo (FMRP-USP) between 2012 and 2022 were evaluated. All patients fulfilled the basic inclusion criteria of having undergone at least two distinct serological tests with positive results for ChD, > 18 years of age, presenting only cardiac abnormalities compatible with ChD, and signing an informed consent to participate in the study.

All included patients had a thorough clinical evaluation that included anamnesis, physical exam, biochemical tests to evaluate diabetes mellitus and dyslipidemia, blood pressure measurement, electrocardiogram (ECG), and assessment of left ventricular ejection fraction (LVEF) through a transthoracic Doppler echocardiogram obtained at rest, using standard methods and also plain chest X-ray examination [11,12].

### Blood collection and DNA extraction

Peripheral blood (5 mL) was collected from each patient before any treatment with benznidazole and added to an equal volume of 6 M guanidine hydrochloride and 0.2 M ethylenediaminetetraacetic acid (EDTA) buffer solution (pH 8.0) [13] for DNA extraction. Guanidine-EDTA blood lysates (GEB) were boiled for 15 min, incubated at room temperature for 24 h, and stored at 4°C until further use [14].

DNA was extracted from 200 μL of GEB samples and eluted with 55 μL of the NucliSens easyMAG system (Biomerieux, France), according to the manufacturer’s instructions.

### Genotyping of Trypanosoma cruzi

Genotyping of *T*. *cruzi* in seven DTUs (TcI-TcVI and Tcbat) was performed based on multilocus conventional polymerase chain reaction (PCR) in association with nested PCR, as described by [15] and modified by [16]. The subsequent identification of genotypes was based on the analysis of the set of profiles of the amplified PCR products presented for each gene target using the following molecular markers (**Table 1**): (1) the intergenic region of the spliced leader gene (SL-IRac) using the UTCC and TCac primers; (2) the intergenic region of the spliced leader (SL-IR) using TCC, TC1, and TC2 primers; (3) the variable D7 domain of the 24Sα rRNA gene, with D75, D76, and D71 primers in semi-nested PCR; and (4) the A10 nuclear fragment in semi-nested PCR, with primers Pr1, P6, and Pr3. The PCR systems, gene targets, and expected sizes of the amplified products are described as in [17].

**Table 1:** Specific primers for *T*. *cruzi* genotyping and diagnosis of Chagas disease.

Genes for genotyping	Primers Name	Sequence
SL-IRac	UTCC	5`- CGTACCAATATAGTACAGAAACTG-3`
	TCac	5`- CTCCCCAGTGTGGCCTGGG-3`
SL-IR I and II	TCC	5`-CCCCCCTCCCAGGCCACACTG-3`
	TCI	5`-GTGTCCGCCACCTCCTTCGGGCC-3`
	TCII	5`-CCTGCAGGCACACGTGTGTGTG-3`
24Sα-rDNA (First round)	D75	5`-GCAGATCTTGGTTGGCGTAG-3`
	D76	5`-GGTTCTCTGTTGCCCCTTTT-3`
24Sα-rDNA (Second round)	D71	5`-AAGGTGCGTCGACAGTGTGG-3`
	D76	5`-GGTTCTCTGTTGCCCCTTTT-3`
A10 (First round)	Pr1	5`-CCGCTAAGCAGTTCTGTCCATA-3`
	P6	5`-GTGATCGCAGGAAACGTGA-3`
A10 (Second round)	Pr1	5`-CCGCTAAGCAGTTCTGTCCATA-3`
	Pr3M	5`-CGTGGCATGGGGTAATAAAGCA-3`
**Primers for diagnosis**	TCZ-F	5`-GCTCTTGCCCACAMGGGTGC-3`
	TCZ-R	5`-CCAAGCAGCGGATAGTTCAGG-3`

**TCZ-F**: *Trypanosoma cruzi*—Forward *primer*; **TCZ-R**: *Trypanosoma cruzi–*Reverse primer.

In all PCR reactions, DNA control samples from reference strains belonging to the six DTUs and Tcbat were used (Colombiana–TcI, Y–TcII, X109/2 –TcIII, CanIII cl1 –TcIV, Bug2148 cl1- TcV, CL Brener–TcVI, and Tcbat 1994—Tcbat), as well as the negative controls and reagents. All amplification reactions were prepared in a final volume of 30 μL, using 12.5 μL of Mastermix Go Taq Green 2X (Promega, Madison, WI, USA), 5 μL *T*. *cruzi* extracted DNA, and primers. The PCR cycling conditions were as described by [15] and were performed using a Thermocycler (G-Storm, model GS 0001). The PCR products were separated by agarose gel electrophoresis (2% or 3% w/v), stained with Midori Green Advanced DNA Stain (Nippon Genetics, Europe GmbH), and viewed on iBright CL 1500 Imaging System. Product size was estimated using a 100 bp molecular marker (Fast Gene Genetics, MWD100).

### Statistical analysis

All experiments were performed with at least two technical replicates. The normality of the data was verified by histograms and Shapiro-Wilk test. Continuous data were expressed as mean ± standard deviation (SD) if normally distributed and as median (interquartile range—IQR) if not normally distributed. Mann-Whitney test was performed to compare LVEF between two independent groups (TcII *versus* other grouped DTU’s). The analysis was conducted using GraphPad Prism (version 7.00) for Windows (GraphPad Software, La Jolla California USA, www.graphpad.com).

Simple and multivariate logistic regression analyses were used to analyze the impact of the *T*. *cruzi* genotype, gender, age and cardiovascular risk factors on the variably severe cases of CCC. Severe cases of CCC were defined as LVEF ≤ 39% [18], and the parasite genotype was separated into two distinct groups: one with only the defined TcII genotype and the other with the other defined and undefined genotypes (TcI, TcIII, TcIV, TcV, and TcVI) or (TcII/TcVI and TcII/TcV/TcVI). Multivariate logistic regression was used to evaluate the impact of sex, age and cardiovascular risk factors as confounders by adjusting the prediction using these variables. The analysis was performed in the R environment (https://www.r-project.org/) using base functions for regression and statistics. The R packages sjPlot was used for data visualization. Statistical significance was set at p < 0.05.

## Results

### Patient characteristics

**Fig 1:**
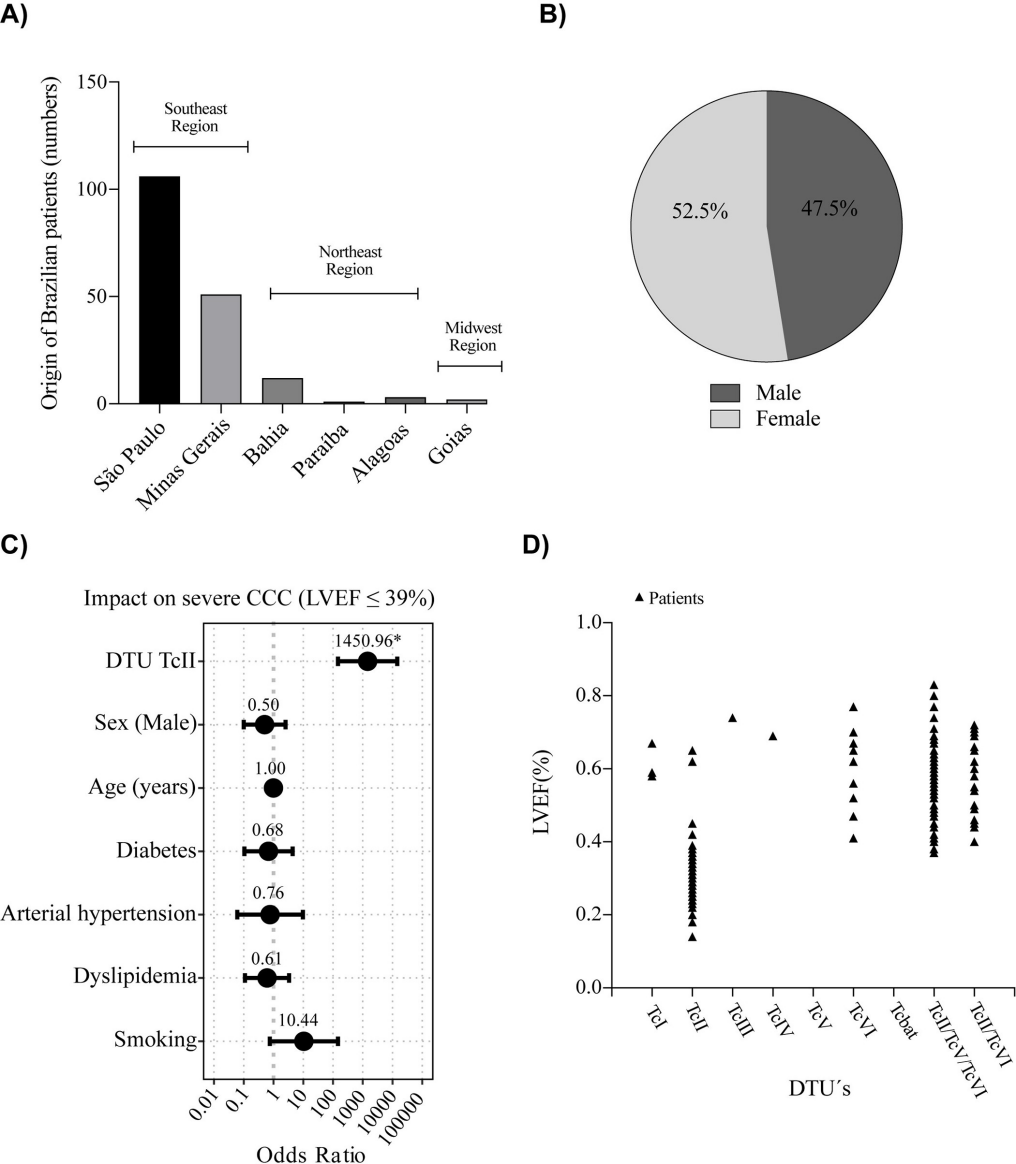
**A)** Geographical origin of each patient (Brazilian States and regions); **B)** Sex Male or Female; **C)** Multivariate logistic regression correlating the impact of gender, age, cardiovascular risk factors and *T*. *cruzi* genotype on the occurrence of severe CCC. **D)** Left ventricular ejection fraction according to different *T*. *cruzi* DTU’s.

All patients (n = 330) were diagnosed by two positive serological tests and qPCR for *T*. *cruzi* **(Table 1)** and were being followed at the Chagas Disease Outpatient Clinic of the Ribeirão Preto Medical School at the University of São Paulo (FMRP-USP) between 2012 and 2022. Of the 330 patient samples evaluated, only 175 tested positive after amplification of the specific genes contained in the *T*. *cruzi* genotyping criteria. The origin of each patient and their gender are shown in **Fig 1A and 1B**. The mean age was 65.2 ± 11. 7 years (range, 24–88 years). Clinical data and cardiovascular risk factors are presented in **Table 2**.

**Table 2:** Clinical parameters, parasite genetics and cardiovascular risk factors present in 175 patients with Chronic Chagas Cardiomyopathy.

	NIHA CLASS				LVEF		CARDIAC PACEMAKER	IMPLANTABLE CARDIAC DEFIBRILLATOR	CARDIOVASCULAR RISK FACTORS			
DTU’s	I	II	III	IV	≤ 39%	> 40%			DIABETES	HYPERTENSION	DYSLIPIDEMIA	SMOKING
**TcI**	1.7% (3/175)	0% (0/175)	0% (0/175)	0% (0/175)	0% (0/175)	1.7% (3/175)	0% (0/175)	0% (0/175)	1.1% (2/175)	1.7% (3/175)	0.5% (1/175)	0.5% (1/175)
**TcII**	16.6% (29/175)	14.8% (26/175)	5.1% (9/175)	0% (0/175)	33.7% (59/175)	2.8% (5/175)	17.7% (31/175)	2.8% (5/175)	8.5% (15/175)	31.4% (55/175)	13.7% (24/175)	6.2% (11/175)
**TcIII**	0.5% (1/175)	0% (0/175)	0% (0/175)	0% (0/175)	0% (0/175)	0.5% (1/175)	0% (0/175)	0% (0/175)	0% (0/175)	0.5% (1/175)	0.5% (1/175)	0% (0/175)
**TcIV**	0.5% (1/175)	0% (0/175)	0% (0/175)	0% (0/175)	0% (0/175)	0.5% (1/175)	0% (0/175)	0% (0/175)	0% (0/175)	0.5% (1/175)	0% (0/175)	0% (0/175)
**TcVI**	5.1% (9/175)	0% (0/175)	0% (0/175)	0% (0/175)	0% (0/175)	5.1% (9/175)	0% (0/175)	0% (0/175)	1.7% (3/175)	4% (7/175)	2.2% (4/175)	0.5% (1/175)
**TcII/TcVI**	13.1% (23/175)	0% (0/175)	0% (0/175)	0% (0/175)	0% (0/175)	13.1% (23/175)	0% (0/175)	0% (0/175)	1.1% (2/175)	9.7% (17/175)	5.1% (9/175)	1.7% (3/175)
**TcII/TcV/TcVI**	42.2% (74/175)	0% (0/175)	0% (0/175)	0% (0/175)	0% (0/175)	42.2% (74/175)	0% (0/175)	0% (0/175)	9.1% (16/175)	35.5% (64/175)	12.5% (22/175)	6.8% (12/175)
**TOTAL**	80% (140/175)	14.8% (26/175)	5.2% (9/175)	0% (0/175)	33.7% (59/175)	66.3% (116/175)	17.7% (31/175)	2.8% (5/175)	21.7% (38/175)	84.5% (148/175)	34.8% (61/175)	16% (28/175)

**DTU’s**: genetic groups; **LVEF**: left ventricular ejection fraction; **NYHA**: New York Heart Association.

All patients with associated cardiovascular risk factors were treated pharmacologically according to the derangements presented (diabetes mellitus, hypertension and dyslipidemia).

### Genotyping of Trypanosoma cruzi

**Fig 2:**
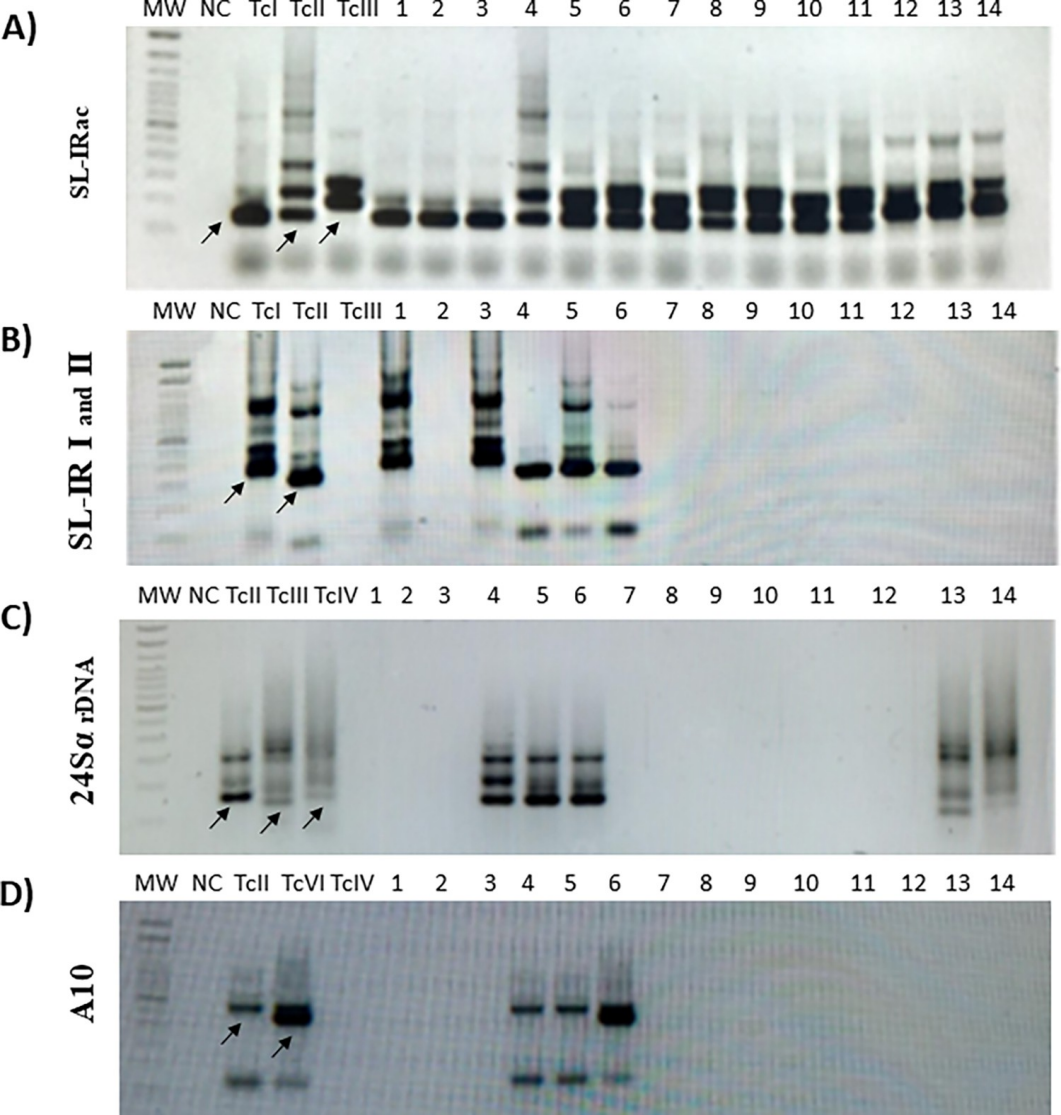
Representative gels of amplified gene products to define *Trypanosoma cruzi* DTU’s. Genes: **A)** the SL-IRac; **B)** the SLIR I and II; **C)** the 24Sα rDNA and **D)** A10. (MW—Molecular Weight marker; NC—Negative control; Positive controls, amplified products of reference strains: TcI: Colombiana; TcII: Y; TcIII: X109/ 2; TcIV: CANIII cl1; TcV: Bug2148 cl1; TcVI: CL Brener. The numbers indicate the code of the sample. Patient’s sample and infecting DTU: 1 –TcI; 2 –TcI; 3 –TcI; 4 –TcII; 5 –TcII; 6 –TcVI; 7,8,9,10,11 and 12 –TcII/TcV/TcVI; 13 –TcIII and 14 –TcIV.

Of the 175 samples in which it was possible to genotype the parasites, 64 showed band profile characteristics of the TcII DTU of *T*. *cruzi*, of which 59 patients had LVEF ≤ 39% (**Figs 1D and 2**). Five samples were infected by the TcII DTU and had LVEF ≥ 40% (**Fig 1D**). In the remaining biological samples (all with LVEF > 40%), the presence of the TcVI genotype was detected in 9 samples, and 23 samples were infected with the TcII/TcVI–(TcII or TcVI) genotype of *T*. *cruzi* (**Figs 1D and 2**). It is worth mentioning that, by convention, the representation of *T*. *cruzi* genotypes interspersed with the symbol (/), indicates that the sample may be infected by any of the DTU’s represented, and that the genotyping criterion was not able to identify a specific DTU. The TcI genotype was identified in three samples, and the TcIII and TcIV genetic profile was detected in only one individual with CCC (**Figs 1D and 2**). The genetic profiles of the TcII/TcV/TcVI—(TcII or TcV or TcVI) genotypes of *T*. *cruzi* were identified in the remaining 74 samples. No mixed infections were identified among the different genotypes of *T*. *cruzi*. (**Figs 1D and 2**).

### Correlation between dtus of *T*. *Cruzi* and severity of chronic Chagas cardiomyopathy

LVEF in TcII genotype was significantly lower in comparison with the other grouped DTU’s: 30% (IQR: 26–36) vs. 59% (IQR: 50–67), respectively, p-value < 0.001 (**Fig 1D**).

Logistic regression confirmed the high and significant impact of the TcII genotype on severe CCC (LVEF ≤ 39%) for both simple regression (odds ratio: 643.10, 95% CI: 121.06–3416.24, p < 0.0001) and after adjustment of sex, age and cardiovascular risk factors by multiple regression (odds ratio: 1450.96, 95% CI: 146.29–14391.32, p < 0.0001) (**Fig 1C**). Despite the increase in the odds ratio in the multiple logistic regression, the impact of sex, age and cardiovascular risk factors on the prediction of severe CCC was not statistically significant, as shown in the forest plot (**Fig 1C**). This was confirmed by analysis of variance (ANOVA) between these models, which revealed no significant improvement compared to the simple model by including sex and age in multiple regression (p = 0.88).

## Discussion

*Trypanosoma cruzi*, the etiological agent of ChD, is composed of heterogeneous subpopulations circulating in both the wild and the domestic cycles [19], and this diversity can be expressed in morphological, biological, antigenic, epidemiological, and genetic aspects [20,21]. Therefore, to better understand the disease, it is important to study the molecular epidemiology of this parasite, which is related to the aforementioned characteristics. Thus, the present study was carried out to identify the genetic strains of *T*. *cruzi* in blood samples isolated from chronic patients managed at the Chagas Disease Outpatient Clinic of the Clinical Hospital of the Ribeirão Preto Medical School (HCFMRP-USP) in an attempt to establish a correlation between the DTUs of the parasite and the severity of ChD cardiomyopathy.

The protocol proposed by [15] was used for molecular genotyping. Of the 330 blood samples from patients with CCC evaluated, only 175 were positive in the amplification of specific genes for genotyping of *T*. *cruzi*.

Of the 175 positive samples, 64 samples of *T*. *cruzi* presented a profile consistent with the TcII genotype, nine with TcVI, three belonging to the TcI genotype, one sample being genetically classified as TcIII and one such as TcIV, 23 as TcII/TcVI, and 74 with the TcII/TcV/TcVI DTU. Of all the patients infected with the TcII genotype, roughly 93% (59/64) had a LVEF ≤ 39% and were considered to have severe cardiomyopathy. For the other DTU’s identified (TcI, TcIII, TcIV, TcVI, TcV/TcVI and TcII/TcV/TcVI), the LVEF was above 39%.

A study conducted in Bahia [22] was the first to use the proposed criteria for the genotyping of *T*. *cruzi* in six DTUs. They identified 18 isolates from domestic cats and vectors in a ChD-endemic region in Bahia, all belonging to the TcII DTU. Our results corroborate those obtained in that study, indicating that this method is efficient for identifying TcII DTU. However, nine samples revealed different patterns or band combinations that could be classified as belonging to the TcVI genotype, as observed in the study conducted by [23,24].

The most important issue that can make the identification of isolates in their seven DTUs very difficult is the occurrence of mixed or polyclonal infections, which have already been documented in several natural and human reservoirs [25], however, fortunately, they were not identified in our samples.

Therefore, the results of this study are in agreement with several studies claiming that TcII strains are mostly associated with human infection, with a predominant geographic distribution between southern and southeastern Brazil [23,26–28]. A classic review covering the ecoepidemiology of different *T*. *cruzi* DTUs [29] pointed out to this distribution. In addition, the TcII DTU was detected in both the domestic and wild cycles of ChD and seems to be closely related to clinical cardiac and digestive manifestations (megacolon and megaesophagus) in human infections [3,29].

In the present study, a strong correlation was observed between the occurrence of severe CCC (LVEF ≤ 39%) and infection with the TcII genotype of *T*. *cruzi*, when compared to the classical cardiovascular risk factors (diabetes mellitus, arterial hypertension, dyslipidemia and smoking), age and sex of the patients. It is important to emphasize that in addition to the aforementioned association, there are several other factors that may be related to the generation of specific clinical manifestations of Chagas disease in human patients, such as: tissue tropism of the strain, the immune response, the habits and quality of life of the host, autoimmunity phenomenon, the presence of molecules that predict prognosis, such as matrix metalloproteinases–MMP [30].

Previous studies [31] recently described the geographic distribution of DTU TcII, and Antigenic diversity of *T cruzi* populations and its effect on the immune response. According to the authors, TcII DTU is present in countries of the southern cone of Latin America (Argentina, Brazil, Chile, Paraguay, and Uruguay), with a higher prevalence in Brazil and Chile. The authors suggested, but did not clearly affirm, the occurrence of indeterminate, cardiac, and digestive forms of ChD associated with this genotype.

Another data of eco epidemiological importance found in our study was the identification of TcI DTU in three patients from southeastern Brazil. TcI DTU is closely related to the sylvatic cycle of ChD, with an almost equal distribution throughout the southern cone, especially in countries in the Amazon region. This genotype has been described infecting human patients who live in or frequent forest regions, considered wild [29,32,33]. TcI is implicated in human diseases in the Amazon, Andes region, Central America, and Mexico [34,35]. Clinical presentations of TcI DTU in humans include Chagas cardiomyopathy and severe meningoencephalitis in immunocompromised hosts [36,37].

Regarding the identification of TcIII DTU in human patients, it was previously shown that the TcIII genotype is related to the sylvatic cycle of ChD in Brazil and adjacent countries, and documented human infections are rare (31). This DTU has occasionally been described in domestic dogs in Paraguay and Brazil, and in peridomestic *Triatoma rubrofasciata* in Rio Grande do Sul, Brazil [38–40]. Consequently, owing to greater investigation and the existence of studies aiming to better understand the ecoepidemiology of circulating DTUs, and knowing that this genotype has been identified in the peridomicile in some regions of Brazil, the possibility of TcIII DTU becoming a source of human ChD cannot be ruled out.

The TcIV genotype occurs in South America, Central America, and North America. It is the second most common DTU cause of Chagas disease in Venezuela and has been reported in outbreaks of oral transmission in Brazil Amazon [29,41].

Regarding the limitations of the study, we can highlight the possibility of degradation of the genetic material stored for long periods in freezing. It is known that specific enzymes have the ability to degrade genetic material even when stored at low temperatures [42], a possible logical explanation for the low positivity of the samples after amplification of the specific genes of the *T*. *cruzi* genotyping criterion.

Studies aimed at understanding the ecoepidemiology of DTU’s involved in human ChD are fundamental to the subsequent progress of research and understanding of the disease processes. These findings of our study can facilitate understanding and better communication among members of the scientific community and establish effective collaboration, particularly among molecular biology laboratories and field research in endemic regions. This type of genetic-clinical correlation approach, without neglecting the its potential relevance for the discovery of new trypanocide drugs, is important for the improvement of control strategies that can reduce the public health burden of ChD in Latin America.

## Conclusions

The results obtained in this study corroborate the data in the literature and demonstrate the predominance of the TcII lineage in a sample of human cardiomyopathy ChD patients from endemic regions of Brazil. This work also demonstrated, in a pioneering way, the existence of a direct relation between the TcII genotype and the most severe clinical cardiac form, in the same sample of chronic ChD cardiomyopathy patients. We also emphasize that these data indicate that it is necessary to constantly search for better biomarkers of clinical evolution, and that treating all individuals infected with *T*. *cruzi* regardless of the infected genotype may indeed still be the best approach to the context. Such therapeutic approach can impact the planning of more effective public health interventions to improve the health of patients with different clinical forms of Chagas disease throughout Latin America.
